# Digital droplet PCR-based quantification of ccfHPV-DNA as liquid biopsy in HPV-driven cervical and vulvar cancer

**DOI:** 10.1007/s00432-023-05077-3

**Published:** 2023-07-14

**Authors:** Fabinshy Thangarajah, Jana Busshoff, Janina Salamon, Marie-Sandrine Pruss, Caroline Lenz, Bernd Morgenstern, Martin Hellmich, Hans Anton Schlößer, Maximilian Lenz, Christian Domröse, Michael R. Mallmann, Peter Mallmann, Jonathan Weiß, Fabian Franzen, Sabine Merkelbach-Bruse, Elke Binot, Marie-Lisa Eich, Reinhardt Büttner, Anne Maria Schultheis, Christina Alidousty

**Affiliations:** 1 Department of Gynecology and Obstetrics, University Hospital of Essen, University of Duisburg Essen, Faculty of Medicine, Essen, Germany; 2 Department of Obstetrics and Gynecology, University of Cologne, Faculty of Medicine and University Hospital Cologne, Cologne, Germany; 3 Institute of Medical Statistics, Informatics and Epidemiology, University of Cologne, Cologne, Germany; 4 Center for Molecular Medicine Cologne and Department of General, Visceral and Cancer Surgery, University Hospital of Cologne, Medical Faculty, Cologne, Germany; 5 Department for Orthopaedic and Trauma Surgery, University Hospital of Cologne, Medical Faculty, Cologne, Germany; 6 Department I of Internal Medicine, Center for Integrated Oncology Aachen Bonn Cologne Duesseldorf, University Hospital of Cologne, Medical Faculty, Cologne, Germany; 7 Institute of Pathology, University Hospital of Cologne, Medical Faculty, Cologne, Germany

**Keywords:** Liquid biopsy, ddPCR, Cervical cancer, Vulvar cancer, cfDNA, cHPV

## Abstract

**Purpose:**

More than 99% of cervical cancers and up to 40% of vulvar cancers are human papillomavirus (HPV) related. HPV 16 and 18 are the most relevant subtypes. Novel technologies allow the detection of minimal amounts of circulating cell-free HPV DNA (ccfHPV-DNA). The aim of this study was to evaluate ccfHPV-DNA assessed by droplet digital PCR (ddPCR) as a biomarker for molecular therapy monitoring in early, advanced, relapsed and metastatic HPV-driven cervical and vulvar cancer.

**Methods:**

Inclusion criteria of the study were histologically proven HPV 16/18-driven cervical and vulvar cancer with first diagnosed disease, newly diagnosed recurrence, or progression of disease. Blood samples were taken pre- and post-therapeutically. Circulating cell-free HPV DNA was quantified using ddPCR and the results were correlated with clinical data.

**Results:**

The mean copy number of ccfHPV-DNA was 838.6 (± 3089.1) in pretreatment and 2.3 (± 6.4) in post-treatment samples (*p* < 0.05). The copy number of ccfHPV-DNA increased with higher FIGO stages (*p* < 0.05), which are commonly used for clinical staging/assessment. Furthermore, we compared the distribution of copy numbers between T-stage 1 versus T-stage 2/3. We could show higher copy number level of ccfHPV-DNA in T-stage 2/3 (*p* < 0.05).

**Conclusions:**

Therapy monitoring with determination of ccfHPV-DNA by ddPCR with a small amount of plasma reflects response to therapy and appears feasible for patients in advanced cancer stages of cervical and vulvar cancer. This promising tool should be examined as marker of therapy monitoring in particular in novel HPV-directed therapies.

## Introduction

More than 99% of cervical cancers and up to 40% of vulvar cancers are human papillomavirus (HPV) related (Zhang et al. 2018; Schnürch et al. 2015). The most frequent HPV subtypes are HPV 16 and 18 (Burd 2003). During the past decades, additional treatment options have improved the outcome for the two cancer types. The GOG 240 Trial showed that addition of the monoclonal antibody bevacizumab to combination chemotherapy in patients with recurrent, persistent, or metastatic cervical cancer is associated with improved survival (Tewari et al. 2014). Especially, immunotherapeutic approaches have been proved to be very effective in the advanced stages of cervical cancer. The Keynote 858 trial demonstrated improved progression-free survival (PFS) and overall survival (OS) by addition of pembrolizumab to chemotherapy ± bevacizumab in patients with recurrent, persistent or metastatic cervical cancer (Colombo et al. 2021).

In addition to clinical examination and imaging of response, serum levels of squamous cell carcinoma antigen (SCC) are currently used for disease monitoring in patients with squamous cell cancer. In patients with HPV-associated adenocarcinoma of the cervix, carcinoembryonal antigen (CEA) or CA 125 are established for the assessment of treatment response. However, the clinical value of serum levels of SCC, CEA and CA125 is limited as these markers are only expressed by a small proportion of patients and can change during the course of the disease. In a previously published study, the sensitivity for SCC was 61.4% for cervical cancer (Holdenrieder et al. 2018). In early-stage adenocarcinoma of the cervix both CEA (range 0.3–219.9 ng/mL) and CA125 (range 2.6–317.4 U/mL) are elevated in in 10.7% (Huang et al. 2020). Monitoring of molecular markers is highly efficient and well established to identify patients with minimal residual disease in hematological cancer, who need intensified treatment. Quantification of HPV in blood of cancer patients using circulating cell-free HPV DNA (ccfHPV-DNA) has been evaluated as biomarker for HPV-driven cancer using quantitative PCR (Wuerdemann et al. 2020). The clinical importance of ccfHPV-DNA has already been analyzed in patients with HPV-related oropharyngeal tumors (Lee et al. 2017; Hanna et al. 2018; Chera et al. 2019). The results of the studies showed a high sensitivity in the detection of tumors and demonstrated the association with the occurrence of relapse and tumor burden (Lee et al. 2017; Hanna et al. 2018; Chera et al. 2019). Furthermore, ccfDNA can also be used to detect genetic alterations (Duffy and Crown 2022). However, the detection rate of ccfHPV-DNA using qPCR is moderate and limits applicability to patients with high tumor burden whereas droplet digital polymerase chain reaction (ddPCR) has a higher sensitivity and has already been applied in patients with oropharyngeal squamous cell carcinoma (OPSCC) (Wuerdemann et al. 2020; Veyer et al. 2020). The detection of ccfHPV-DNA in OPSCC as a marker for residual disease and therapy monitoring has been investigated (O’Boyle et al. 2022; Haring et al. 1214). Interestingly, ccfHPV-DNA levels are known to be associated with residual disease in patients with HPV + OPSCC undergoing curatively intended surgery (O’Boyle et al. 2022; Haring et al. 1214). Furthermore, longitudinal changes of ccfHPV-DNA correlate with treatment response and ccfHPV-DNA elevations are observed earlier than progress in conventional imaging (O’Boyle et al. 2022; Haring et al. 1214). Only few studies investigated ccfHPV-DNA using ddPCR in gynecologic cancer (Cheung et al. 2019; Jeannot et al. 2016, 2021; Cabel et al. 2021). The aim of this study was to evaluate ccfHPV-DNA using ddPCR as a biomarker for molecular therapy monitoring in HPV-driven cervical and vulvar cancer.

## Materials and methods

### Patients

Inclusion criteria of the study were histologically proven HPV 16/18-driven cervical and vulvar cancer. Patients of any stage with first diagnosed disease, newly diagnosed recurrence or progression of disease were eligible. Blood samples were obtained pre- and post-therapeutically. Imaging was performed before treatment for all patients. The study had been approved by the local ethics committee (19–1367).

### Molecular diagnosis of HPV infection

HPV status was determined using the HPV 3.5 LCD-Array Kit (Chipron, Berlin, Germany) according to the manufacturer’s instructions.

### Blood collection and plasma preparation

Whole blood of all patients with HPV-induced cervical cancer was collected at different time points during the patient’s follow-up and transferred to PAXgene^®^ Blood ccf DNA tube (Qiagen, Hilden, Germany). After blood draw, tubes were immediately inverted 10 times and stored at room temperature to a maximum of 5 days until further processing.

For plasma preparation, the PAXgene^®^ Blood ccf DNA tubes were centrifuged at 1.600×*g* for 10 min at room temperature using a swing bucket. A total of 2–4 ml of plasma of each sample was aspirated and transferred to 2 ml DNA LoBind Tubes (Eppendorf, Hamburg, Germany). To remove any residual blood cells, the supernatant was centrifuged at 16.000×*g* for 10 min at 4 °C using a fixed angle rotor. The supernatant was transferred again to 2 ml DNA LoBind Tubes (Eppendorf). Plasma was stored at − 80 °C until ccfDNA extraction.

### Extraction of circulating cfDNA and analysis by ddPCR

Plasma samples were thawed and immediately processed using the Maxwell^®^ RSC ccfDNA Kit (Promega, Madison, WI) according to the manufacturer’s instructions. After elution, DNA was stored at 4 °C until the next day.

For ddPCR, Bio-Rad QX200 Droplet Digital PCR system was used according to the manufacturer’s instructions (Instruction Manual, QX200™ Droplet Generator, Bio-Rad Laboratories, Hercules, California, USA). Briefly, 10 ng of DNA used as positive control, 5 ng DNA used as negative control or 5 µl cfDNA isolated from plasma were mixed with ddPCR supermix for probes (no dUTPs, Bio-Rad laboratories) and primer/probe set (Bio-Rad Laboratories). Droplets were generated using 20 µl of the reaction mixture and 70 µl of droplet generation oil. For positive controls, DNA isolated from Caski cells harboring an intact HPV-16 genome was used. HT29 cells were used as negative control. A no-template control was inserted to monitor contamination. The housekeeping gene GAPDH was used to ensure DNA integrity for each sample. All samples (except controls) were run and analyzed in triplicates.

All primers and probes were ordered through Bio-Rad, with the following Assay ID/Sequences: GAPDH Assay ID: dHsaCNS794216737; HPV 16 forward primer: TCCAGCTGGACAAGCAGAAC, HPV 16 reverse primer: CACAACCGAAGCGTAGAGTC, HPV 16 probe: ACAGAGCCCATTACAAT, HPV 18 forward primer: AACATTTACCAGCCCGACGA, HPV 18 reverse primer: TCGTCTGCTGAGCTTTCTAC, HPV 18 probe: AACCACAACGTCACACAA.

The absolute quantity of DNA per sample (copies/µl) was assessed using QuantaSoft software (v1.7.4.0917, Biorad).

### Statistical analysis

First, a descriptive analysis of patients’ characteristics was performed. Data are presented as mean ± standard deviation (SD) or count (percentage), respectively. Statistical analyses and figures were performed using SPSS 28.0 (IBM) and GraphPad Prism V.9.0.2 (GraphPad, USA). Significant differences were calculated using the nonparametric, unpaired and two-tailed Mann–Whitney test for unpaired comparisons and the Wilcoxon matched-pairs signed rank test for paired comparisons of pre- and post-therapeutic samples. *p* values < 0.05 were considered as significant.

## Results

A total of 19 patients, 15 with cervical and 4 with vulvar cancer, could be included into this study. Main characteristics are summarized in Table 1. Overall, 15/19 patients had first diagnosed cervical or vulvar cancer, whereas 4/19 patients had a recurrence of disease. Sixteen patients had a diagnosis of squamous cell carcinoma, and 3 had adenocarcinomas. Mean age of the patients was 49.6 (± 12.7) years.

**Table 1:** Patient characteristics

	*n*	Percentage
Total	19	100
First diagnosed disease (*n*)	15	78.9
Recurrent disease	4	21.1
Adenocarcinoma	3	15.8
Squamous cell carcinoma	16	84.2
HPV type		
16	16	84.2
18	2	10.5
16 + 18	1	5.3
Nicotine abuse		
Yes	5	26.3
No	14	73.7
Figo-stage		
I	10	52.6
II	1	5.3
III	3	15.8
IV	1	5.3
N/A (rec. disease)	4	21.1
T-stage		
I	9	47.4
II	3	15.8
III	3	15.8
N/A (rec. disease	4	21.1
N-stage		
0	12	63.2
1	3	15.8
N/A (rec. disease)	4	21.1
M		
0	17	68.4
1	2	5.3
G		
1	0	0
2	13	68.4
3	3	15.8
x	3	15.8
L		
0	10	52.6
1	5	26.3
x	4	21.1
V		
0	13	68.4
1	1	5.3
x	5	26.3

Recurrent disease (rec. disease)

16/19 (84.2%) had an HPV 16-associated disease; whereas, 2/19 (10.5%) had an HPV 18-associated disease; one patient had a simultaneous infection with HPV 16 and 18. The detection rate for ccfHPV-DNA in pretreatment samples was 63.2% in the whole cohort and 53.3% within the group of patients with first diagnosed cervical or vulvar cancer. The detection rate of the conventional tumor marker SCC in serum samples from patients with squamous cell carcinoma of the vulva or with cervical cancer was 43.7% in the whole cohort and 38.5% with first diagnosed disease, respectively.

**Fig. 1:**
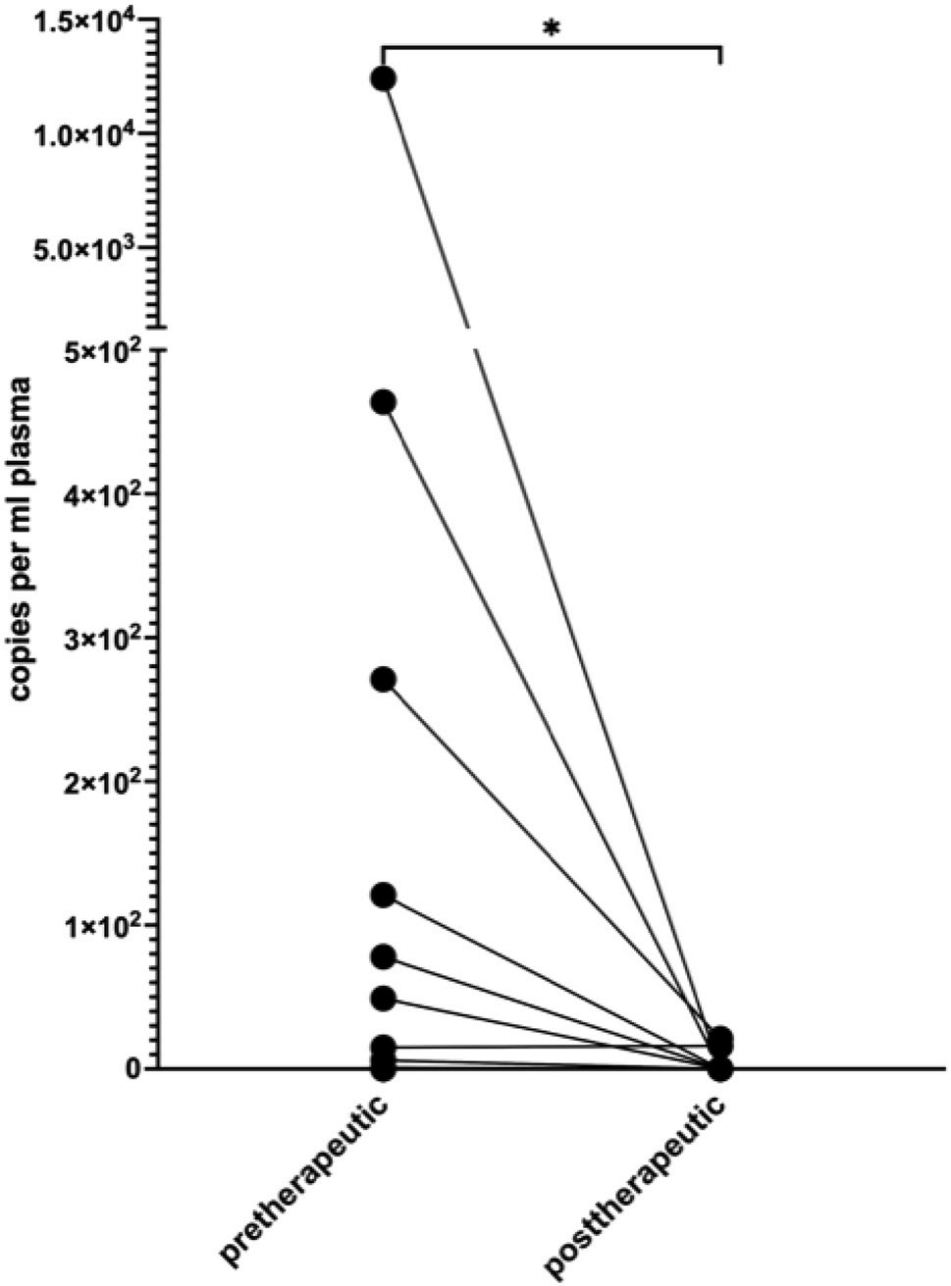
Illustration of pre- and post-therapeutic ccfHPV-DNA level (copies per ml plasma). Wilcoxon matched-pairs signed rank test for paired comparisons of pre- and post-therapeutic samples. Significant difference is indicated by asterisks. *p ≤ 0.05, **p ≤ 0.01, ***p ≤ 0.001, ****p ≤ 0.0001

**Fig. 2:**
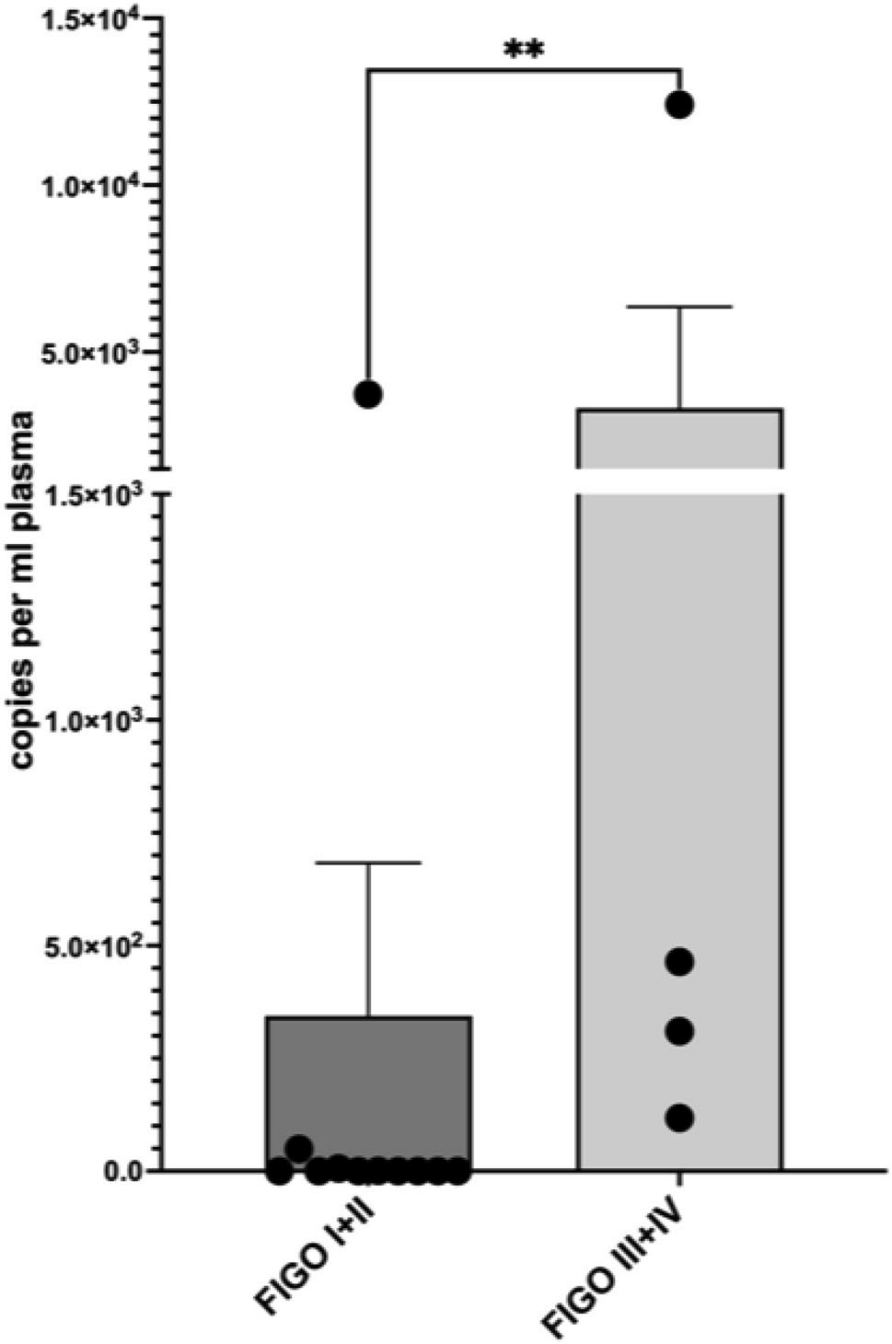
Comparison of mean copy number ccfHPV-DNA level per ml plasma between Figo stages I/II versus Figo stages III/IV. Significant differences were calculated using the nonparametric, unpaired and two-tailed Mann–Whitney test for unpaired comparisons. Significant difference is indicated by asterisks. *p ≤ 0.05, **p ≤ 0.01, ***p ≤ 0.001, ****p ≤ 0.0001

**Fig. 3:**
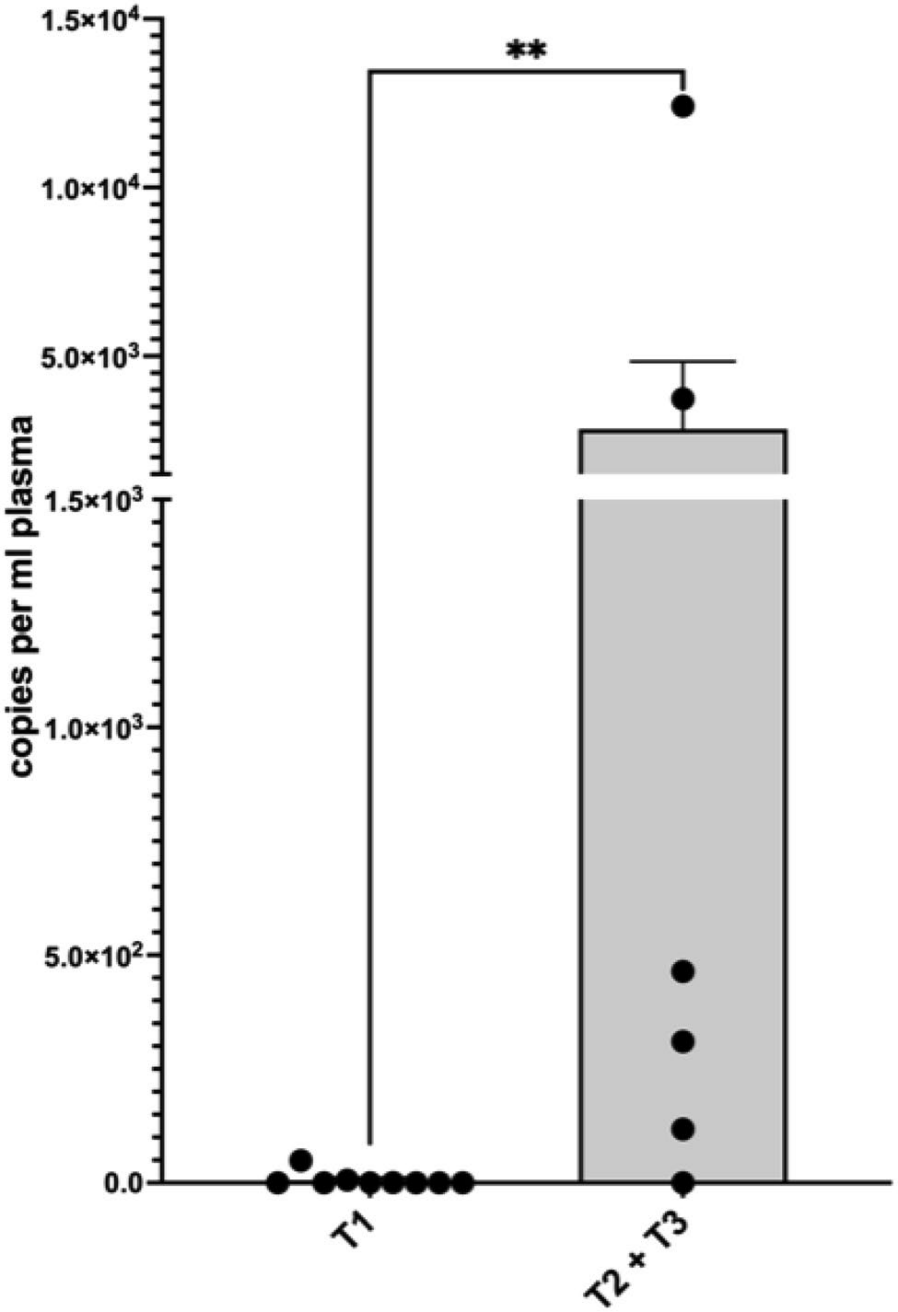
Comparison of mean copy number ccfHPV-DNA per ml plasma between T1 versus T2/3. Significant differences were calculated using the nonparametric, unpaired and two-tailed Mann–Whitney test for unpaired comparisons. Significant difference is indicated by asterisks. *p ≤ 0.05, **p ≤ 0.01, ***p ≤ 0.001, ****p ≤ 0.0001

For patients with a first diagnosis of cervical or vulvar cancer, the mean copy number was 1139.5 (± 3260.8) per ml plasma. In our cohort, none of the patients with first diagnosed cervical- or vulvar cancer had residual ccfHPV-DNA after primary therapy. In 16 patients, we were able to compare pre- and post-therapeutic copy number of ccfHPV-DNA and observed a drop of the mean copy number from 838.6 (± 3089.1) in pretreatment compared to a 2.3 (± 6.4) in post-treatment samples. The difference in copy number levels of ccfHPV-DNA between these timepoints was statistically significant (*p* = 0.013) (Fig. 1). We further analyzed a relation between stages and the copy number of ccfHPV-DNA per ml plasma. Stratification of patients according to FIGO stages revealed a copy number of 344.3 (± 1123.3) in patients with FIGO stage I/II compared to 3326.25 (± 6059.49) in patients with FIGO stage III/IV (*p* < 0.05). The copy number of ccfHPV-DNA increased with higher FIGO stages (Fig. 2). Additionally, we compared the mean copy number of ccfHPV-DNA between T-stage 1 which was 6.2 (16.2) to the mean copy number in T-stage 2/3, which was 2839.3 (± 4898.1) (*p* < 0.05). The copy number of ccfHPV-DNA increases with higher T-stages (Fig. 3).

**Fig. 4:**
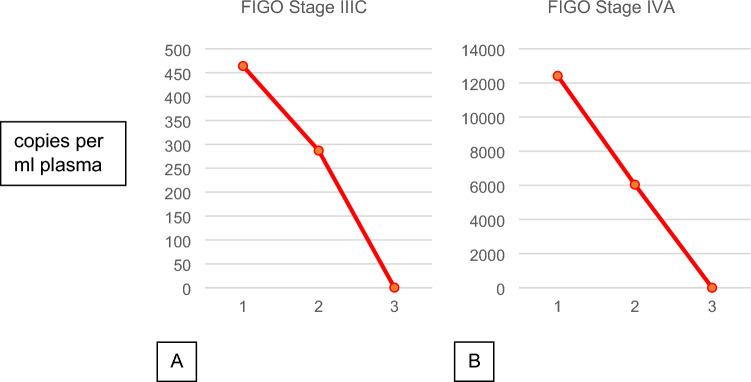
Dynamics of ccfHPV-DNA during therapy of first diagnosed cervical cancer. This figure shows patients with FIGO IIIC stage (**A**) und FIGO IVA stage (**B**) cervical cancer. It shows the copy number at baseline (1), after lymphadenectomy (2) and induction chemotherapy followed by radiochemotherapy (3). In both patients, ccfHPV-DNA was not detectable after the therapy

From two patients, multiple samples matched to different steps of the therapy could be collected and allowed to compare the evolution of ccfHPV-DNA levels during the course of therapy. Both patients underwent an individualized therapeutic concept based on interdisciplinary board decision. One patient was first diagnosed with locally advanced squamous cell carcinoma of the cervix, FIGO IIIC (Fig. 4A). At baseline, the copy number was 464 per ml plasma. The patient underwent a laparoscopic lymphadenectomy (LAD) with resection of bulky nodes. After the LAD, the copy number of ccfHPV-DNA decreased to 287 per ml plasma. In addition, the patient received induction of chemotherapy and chemoradiation and the copy number of ccfHPV-DNA decreased to 0 copies/ml plasma after completion of these therapies. Figure 4B shows the dynamics of a patient with FIGO IVA cervical cancer who underwent laparoscopic lymphadenectomy first and then underwent induction of chemotherapy followed by chemoradiation.

## Discussion

In this cohort of patients with cervical and vulvar cancer, we were able to show that in 63.5% of the cases, HPV-derived DNA was detectable in the pretreatment plasma samples. Previous studies showed detection rates between 61.6 and 100% depending on tumor stage (Cheung et al. 2019; Cabel et al. 2021; Kang et al. 2017).

Cabel et al. reported that ccfHPV-DNA detection before chemoradiation was associated with tumor stage and lymph node status (Cabel et al. 2021). We were able to confirm the significant correlation between tumor stage and ccfHPV-DNA (*p* < 0.05). Furthermore, our data showed a correlation between ccfHPV-DNA and FIGO stage in patients with first diagnosed cervical cancer and vulvar cancer (*p* < 0.05), which is in line with previously published data (Jeannot et al. 2016). Especially, these results indicate the association between tumor burden and the level of detected ccfHPV-DNA.

In our cohort, none of the patients had residual ccfHPV-DNA after adjuvant therapy. Jeannot et al. reported that patients with persistent ccfHPV-DNA in serum relapsed within a median time of 10 months (range 2–15) from ccfHPV-DNA detection (Jeannot et al. 2021). In current publications, ccfDNA determination with ddPCR has been analyzed as a prognostic factor. It is known that high levels of ccfDNA are associated with increased risk of relapse (Cheung et al. 2019). We are currently addressing this aspect in an ongoing part of this study. The comparison of pre- and posttherapeutical determination of ccfHPV-DNA showed a significant decrease of copy numbers, which shows the ability of this marker to monitor therapeutic effects and tumor mass reduction. This observation can be made in the group of first diagnosed patients as well as in the group of patients receiving therapy due to relapse or progressive disease. Our data from two patients with diagnostic staging lymphadenectomy with resection of bulky nodes, moreover, suggest that a limited tumor mass reduction can probably be detected with ccfHPV-DNA. The copy number of ccfHPV-DNA was decreased after lymphadenectomy in both cases. Monitoring of response by liquid biopsies with ccfHPV-DNA appears very promising in cervical and vulvar cancer and may improve clinical staging, if confirmation of our results and additional standardization is achieved in larger cohorts.

Monitoring of ccfHPV-DNA could be useful in several clinical scenarios. First, therapeutic vaccination targeting HPV 16 E6 is currently evaluated in clinical trials with promising early results (Youn et al. 2020; Bakker et al. 2021). Examination of ccfHPV-DNA could represent a marker to specifically monitor therapeutic effects of HPV-directed agents in this setting. Another important aspect is whether the quantification of ccfHPV-DNA after therapy can help to improve therapeutic algorithms. In other tumor entities, e.g., oropharyngeal cancer, it has been discussed whether the detection of ccfHPV-DNA could identify patients with a partial response who would potentially benefit from salvage therapies (Li et al. 2023). The use of ccHPV-DNA for risk stratification could also be an interesting approach for patients with cervical cancer. According to the current German guidelines for the treatment of cervical cancer, adjuvant radiation is recommended in the presence of risk factors (e.g., lymph node invasion, residual disease, neuroendocrine tumor, tumor size > 4 cm or G3 in combination with two other risk factors) (S3-Leitlinie Diagnostik, Therapie und Nachsorge der Patientin mit Zervixkarzinom 2012). Minimal residual disease (MRD) is a common parameter guiding therapeutic decisions in leukemia (Slade et al. 2023) and MRD detected by ccfHPV-DNA could be applied for risk stratification and identification of patients, who need intensified adjuvant therapy in vulvar or cervical cancer. The detection of minimal residual disease in other tumor entities is often based on the detection of somatic mutations (Kasi et al. 2022), which can be of variable importance. For example, only somatic mutations, which are not associated with clonal hematopoiesis are of prognostic value in leukemia (Jongen-Lavrencic et al. 2018). Accordingly, analyses of clonal evolution to detect relapse, to identify non-responders of therapy and to guide molecular targeted therapies are currently evaluated in metastatic colorectal cancer (Wong et al. 2023). Changes in DNA methylation are common in tumor tissue and can also be detected in plasma (Kasi et al. 2022; Luo et al. 2020) and results from recently published studies indicate that DNA methylation holds promise for improved MRD detection in different types of cancer (Kasi et al. 2022; Fu et al. 2018; Murray et al. 2018; Musher et al. 2020; Taieb et al. 2021). The integration of HPV DNA into the host DNA is involved in the carcinogenesis of vulvar and cervical cancer and is not detectable in normal tissue. This tumor specificity of ccfHPV-DNA represents a huge advantage of the method presented in this trial. Our approach should be further analyzed in prospective clinical trials with patients with HPV-related gynecologic tumor.

## Conclusion

In conclusion, we demonstrate that the detection of ccfHPV-DNA by ddPCR is feasible in cervical and vulvar cancer. It appears highly promising for therapy monitoring in advanced cancer stages, as it reflects response to therapies and copy numbers are related to tumor burden. It should be evaluated in prospective clinical trials as marker of therapy monitoring or MRD-guided therapeutic algorithms. It is of particular interest for novel HPV-directed therapies.

## Data Availability

Not applicable.
